# Yoga and Rehabilitation: Physical, Psychological, and Social

**DOI:** 10.1155/2013/624758

**Published:** 2013-11-24

**Authors:** Shirley Telles, Elisa Kozasa, Luciano Bernardi, Marc Cohen

**Affiliations:** ^1^Patanjali Research Foundation, Haridwar, India; ^2^Hospital Israelita Albert Einstein, 05651-901 São Paulo, SP, Brazil; ^3^Department of Psychobiology, Universidade Federal de São Paulo, 04023-062 São Paulo, SP, Brazil; ^4^Department of Internal Medicine, University of Pavia, Italy; ^5^Folkhälsan Research Center, University of Helsinki, Finland; ^6^Royal Melbourne Institute of Technology, Melbourne, VIC, Australia

Yoga was intended for spiritual evolution [1], but increasingly nowadays it is used for its incidental benefits such as stress reduction and managing lifestyle related disorders. Despite its Indian origin, the number of yoga practitioners in Western society is growing: in the United States, for example, a national survey showed that 6.1% of the adults were practicing yoga in 2007 [2].

Apart from the benefits of Yoga practice in preventing and managing disease, yoga has several applications in rehabilitation (*rehabilitare* = to restore, in Latin). Rehabilitation is of different types such as (i) physical, (ii) psychological, and (iii) social. Yoga, as a way of life, has helped persons with physical disorders to return to health, an example being coronary artery disease [3, 4]. Other conditions which have benefitted from yoga practice include stroke after cerebrovascular accidents [5] and patients with heart failure, in whom exercise capacity, oxygen saturation, and parasympathetic activity were restored [6, 7]. Yoga breathing or *pranayama* was especially beneficial for COPD [8]. Practicing yoga has also been used with good results in degenerative disorders such as idiopathic Parkinson's syndrome [9] and muscular dystrophy [10].

With regard to psychological rehabilitation, yoga practice has helped restore the psychological function and mental equilibrium in persons with posttraumatic stress disorder [11] and even certain psychotic conditions [12].

Finally, yoga practice can help people who are at a disadvantage because of their social circumstances. This includes persons in jail [13], those from the “inner city” [14], children in remand homes [15, 16], and older people living in community centers [17, 18]. Social rehabilitation includes dimensions of physical and psychological rehabilitation. 

Despite the research cited above, there is a continued need for research. A summary of yoga reviews pointed to the importance of larger-scale, rigorous research with higher methodological quality and adequate control groups.

Additionally, it should be stressed that although research should be aimed at yoga as a whole (hence including postures as well as meditation, respiratory practices, diet, and other aspects) this is obviously very complex, as the contribution of many different aspects cannot be easily identified, and confounding factors may play a decisive role. Also, the complexity and variety of different yoga techniques make a comparison between different studies very difficult. On the other hand, a “reductionist” approach seems to be more feasible from a scientific point of view, due to an easier separation of the specific effects of a single intervention (e.g., the analysis of one posture or the analysis of a single type of *pranayama*), but it is obviously at the risk of missing the whole effect of yoga. As a typical example, if one measures only the energy expenditure of a single yoga session, one may conclude that yoga will be of no use to lose weight, but other crucial effects of yoga related to weight control could be missed, such as the attention to diet and possibly a direct effect of *pranayama* on the hypothalamus, through which yoga does actually lead to weight loss. Thus, a careful balance between these opposite aspects needs to be taken into account when planning research on yoga. 

 The analyzed reviews suggested a number of areas where yoga may be beneficial, but more research is required for them to really establish such benefits. Nevertheless, some meta-analyses indicated that there are several randomized clinical trials of relatively high quality indicating beneficial effects of yoga for pain-associated disability and mental health [8]. Considering the large number of different yoga techniques and schools of yoga, it is also important for researchers to describe, in detail, the specific method used in a given study.

Yoga is worth investigating as it is relatively cost-effective, its practices can be adapted for different groups of patients, and if well oriented, the risks of side effects are very low. We hope that this special issue will give new insights for the development of novel, well-designed studies in the field of yoga and rehabilitation.

*Shirley Telles* *Shirley Telles* *Elisa Kozasa* *Elisa Kozasa* *Luciano Bernardi* *Luciano Bernardi* *Marc Cohen* *Marc Cohen*
